# Supplementary figures and images for: In search of autophagy biomarkers in breast cancer: Receptor status and drug agnostic transcriptional changes during autophagy flux in cell lines
Source: PLoS One. 2022 Jan 6;17(1):e0262134. doi: 10.1371/journal.pone.0262134 (PMC8735604; doi:10.1371/journal.pone.0262134)

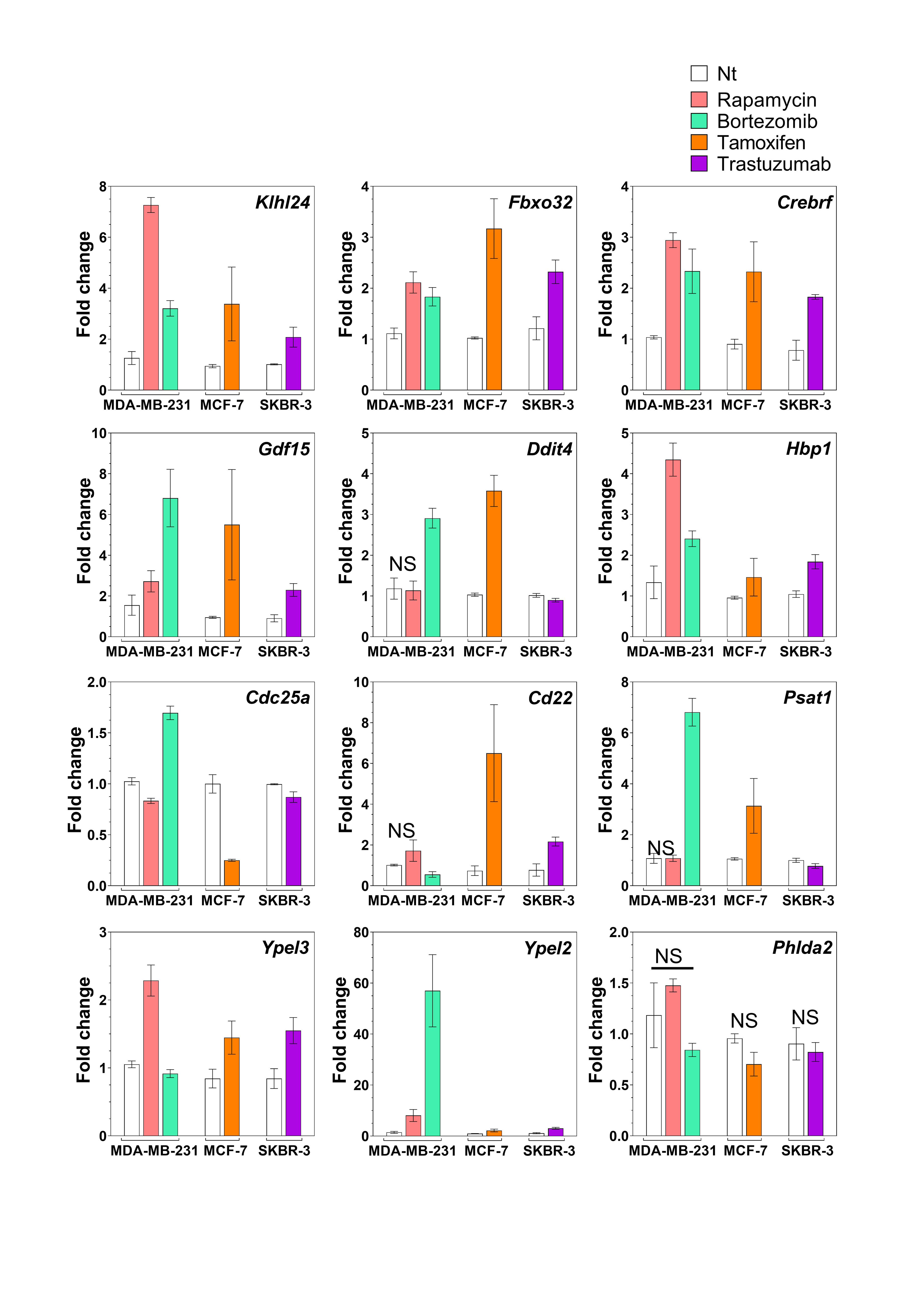

Supplement: S1 Fig — Relative expression of the 12 mRNAs plotted as fold change over the non-treated for each drug and cell line. All the comparisons between untreated and drug treated within the same cells are significant with p ≤ 0.05 except when labeled with NS = non-significant (p ˃ 0.05). (TIF) [file pone.0262134.s001.tif]

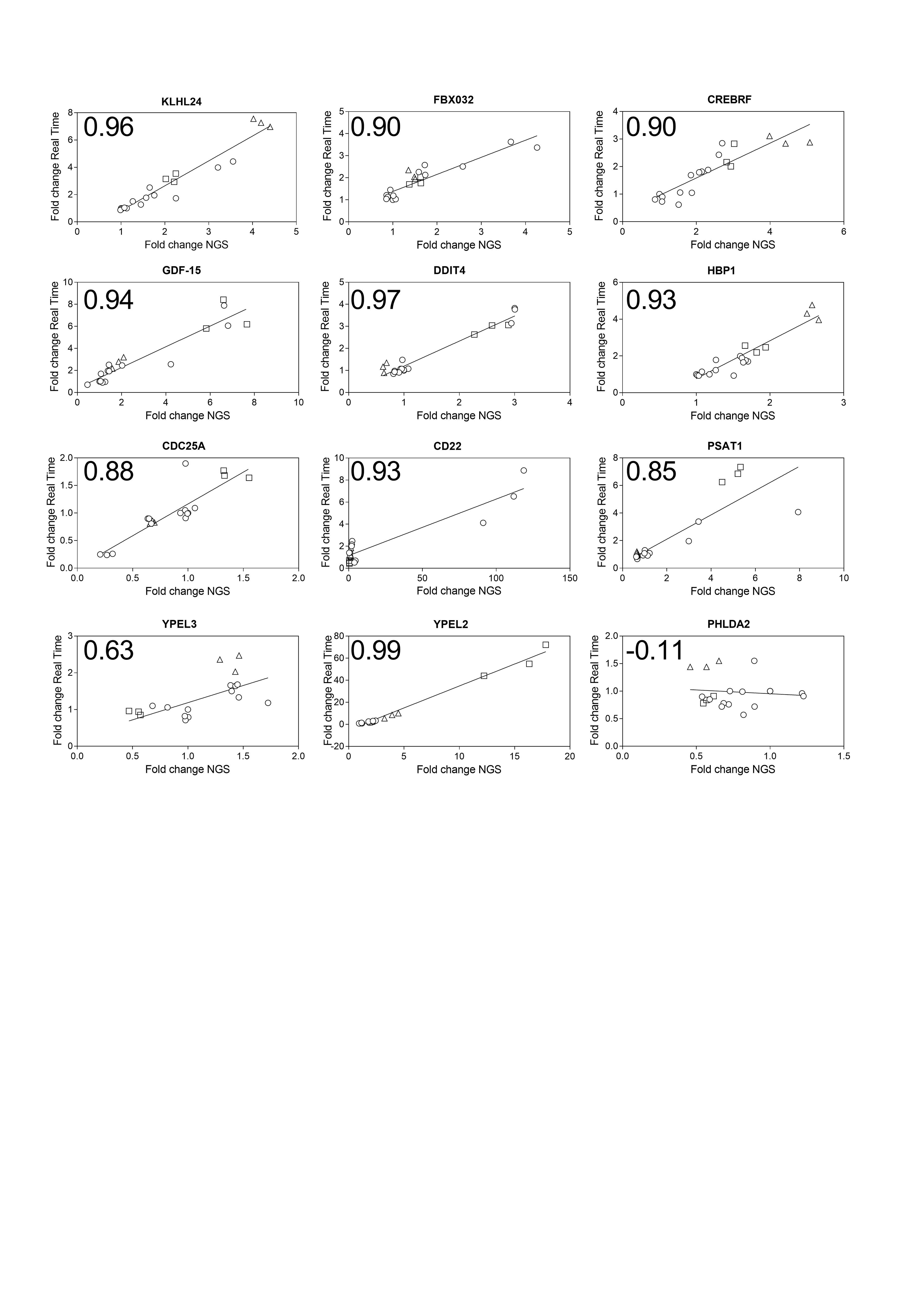

Supplement: S2 Fig — Correlation graphs with fold change data from Q-RT-PCR on Y axis and fold change data from the RNA sequencing data (NGS) on X axis. The numbers in the top left corner represent the Pearson r value. (TIF) [file pone.0262134.s002.tif]

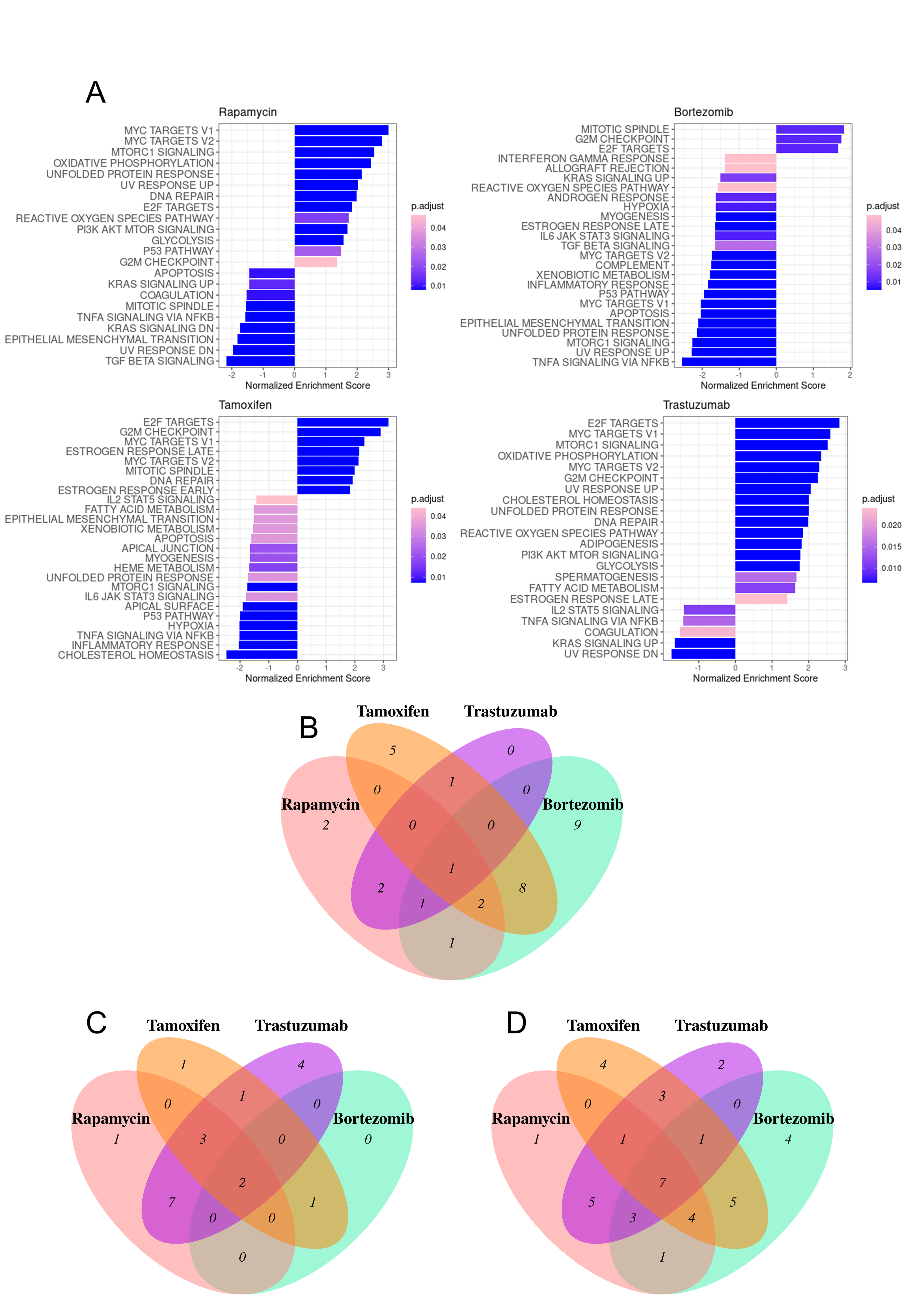

Supplement: S3 Fig — A) GSEA normalized enrichment score for each hallmark gene set in rapamycin, bortezomib, tamoxifen and trastuzumab treated cells. Positive normalized enrichment scores indicate gene sets that are positively enriched in each treatment compared to control. Gene sets colored in darker blue indicate higher statistical significance. Only sets with FDR < 0.05 are included in the results. B) Venn diagram showing the overlap of significantly underrepresented hallmark gene sets between rapamycin (pink), bortezomib (green), tamoxifen (orange) and trastuzumab (purple) treated cells. C) Venn diagram showing the overlap of significantly overrepresented hallmark gene sets between drug treatments. D) Venn diagram showing the overlap of significantly correlated hallmark gene sets between drug treatments. (TIF) [file pone.0262134.s003.tif]

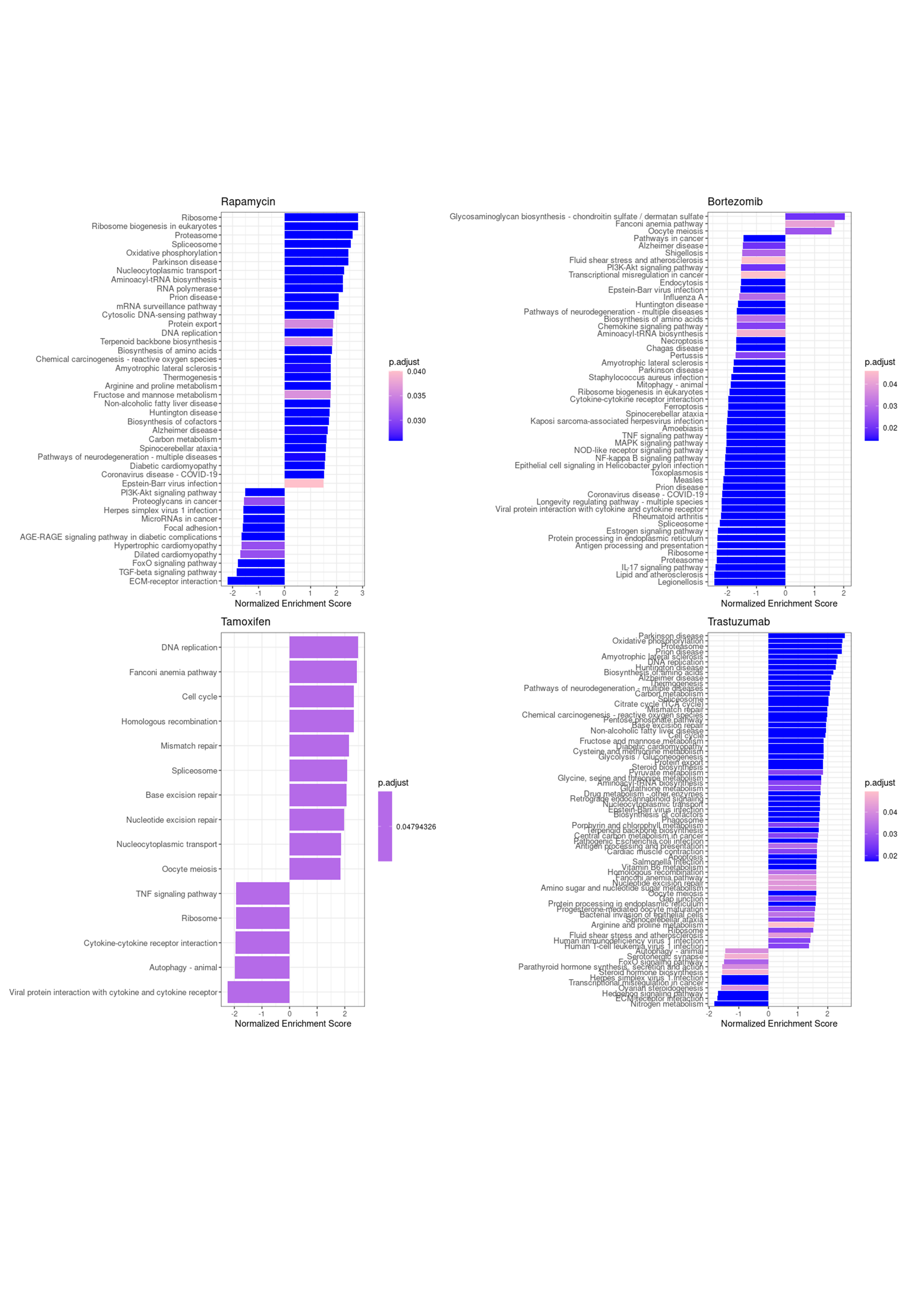

Supplement: S4 Fig — GSEA normalized enrichment score for each KEGG pathway in rapamycin, bortezomib, tamoxifen and trastuzumab treated cells. Positive normalized enrichment scores indicate pathways that are positively enriched in each treatment compared to control. Gene sets colored in darker blue indicate higher statistical significance. Only pathways with FDR < 0.05 are included in the results. (TIF) [file pone.0262134.s004.tif]

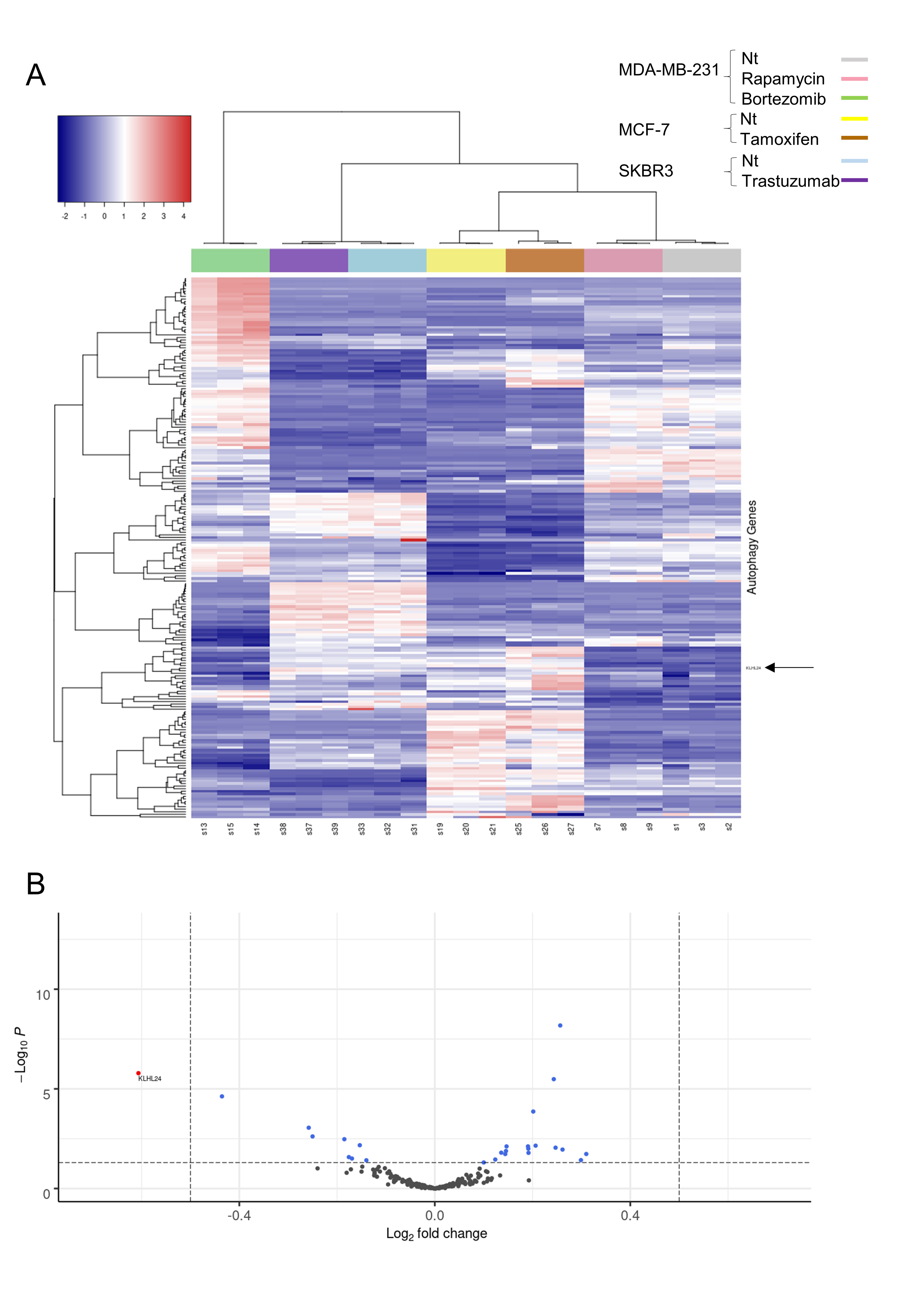

Supplement: S5 Fig — A) Hierarchical clustering heatmap of gene expression values for the list of 232 autophagy-related genes from the Human Autophagy Database in RNASeq data from untreated and treated breast cancer cell lines. The arrow indicates Klhl24 mRNA. B) Volcano plot for 232 autophagy genes from the Human Autophagy Database. Red and blue colored dots show genes that pass the P-value threshold of <0.05 (horizontal line). The vertical lines are log2 fold change values <-0.5 and >0.5 (red dot). (TIF) [file pone.0262134.s005.tif]

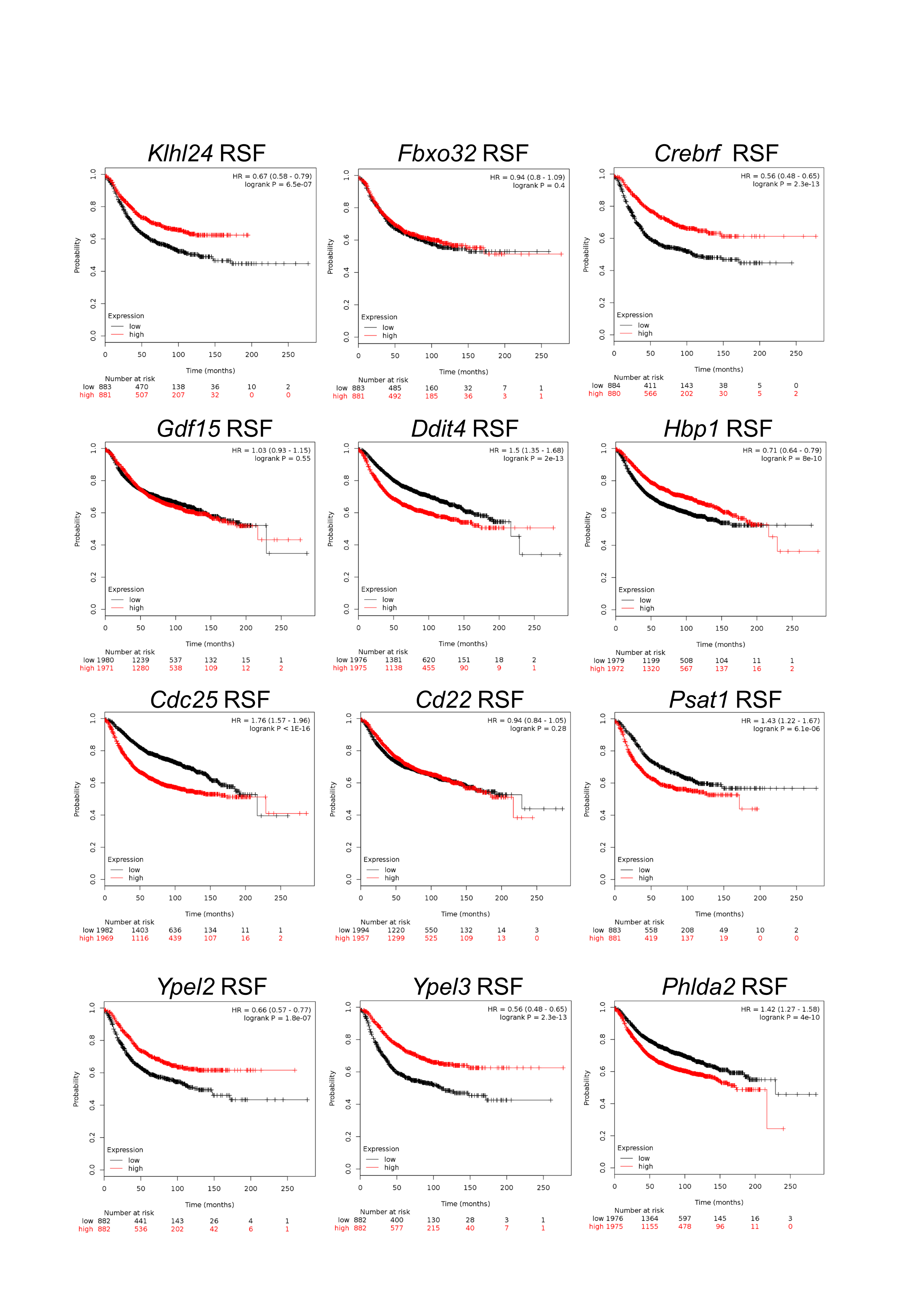

Supplement: S6 Fig — High (red line) or low (black line) levels of each gene are correlated with patient’s outcome with KM Plotter software. P values are in the top right of each graph. (TIF) [file pone.0262134.s006.tif]

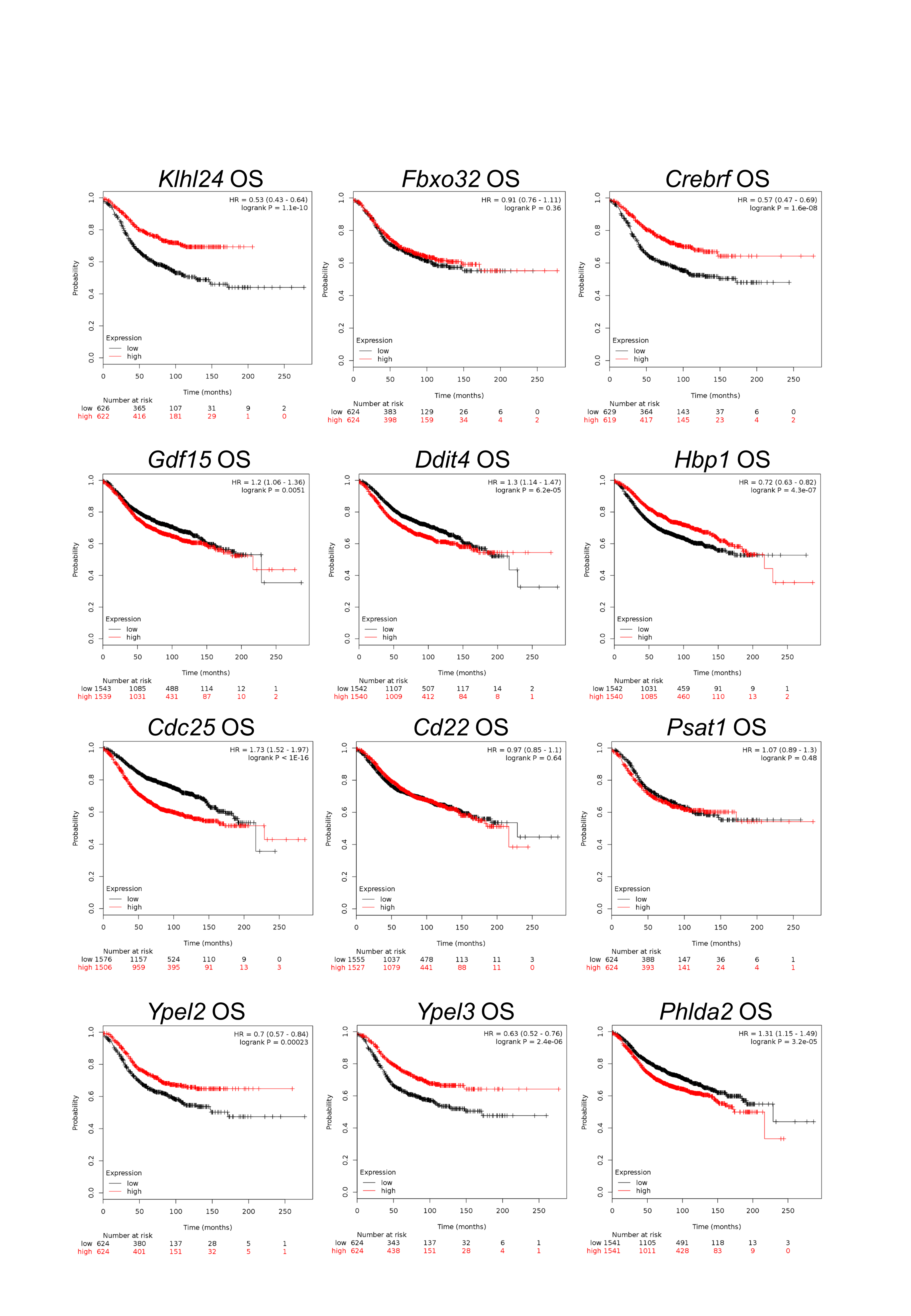

Supplement: S7 Fig — High (red line) or low (black line) levels of each gene are correlated with patient`s outcome with KM Plotter software. P values are in the top right of each graph. (TIF) [file pone.0262134.s007.tif]

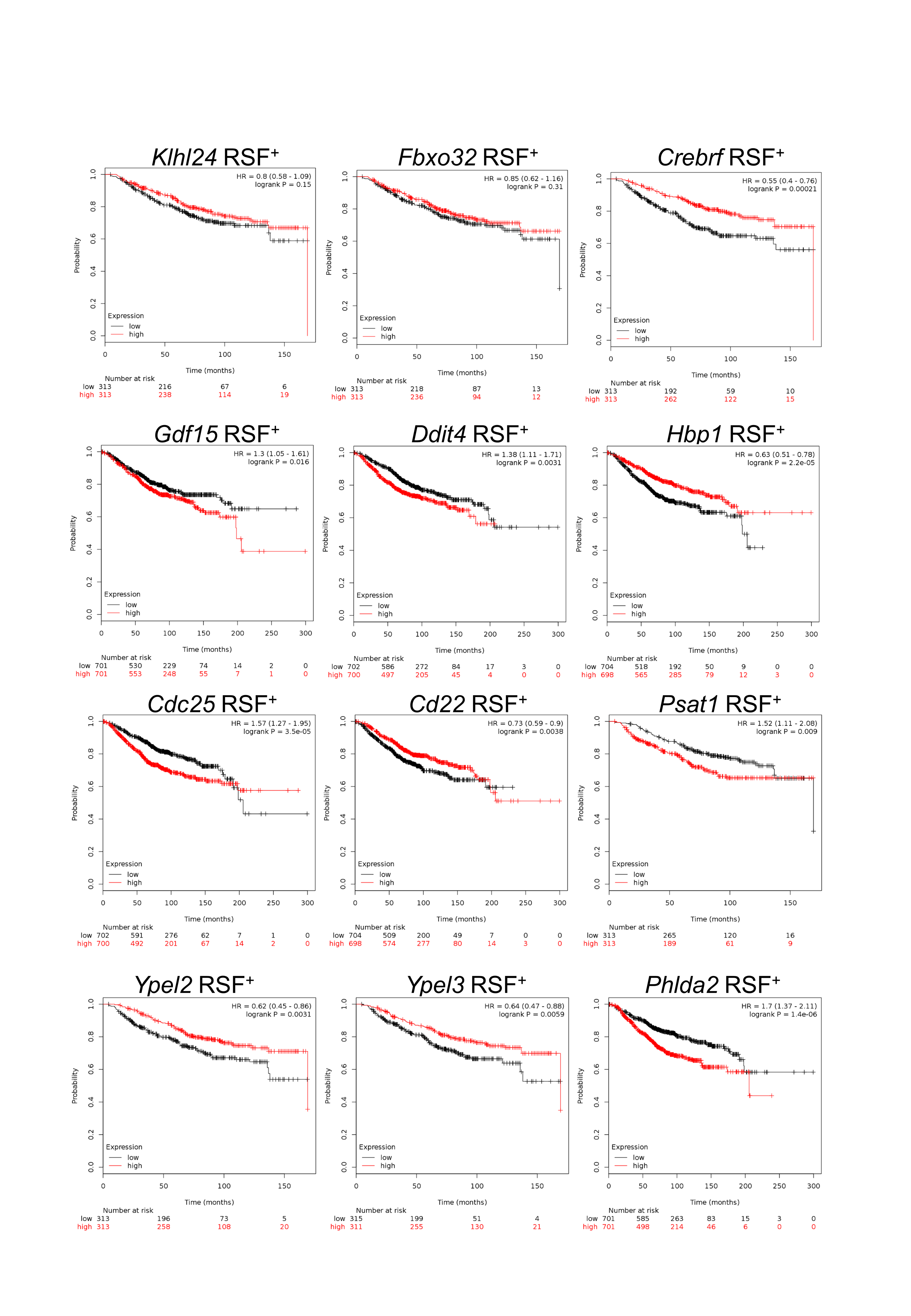

Supplement: S8 Fig — High (red line) or low (black line) levels of each gene are correlated with patient`s outcome with KM Plotter software. P values are in the top right of each graph. (TIF) [file pone.0262134.s008.tif]

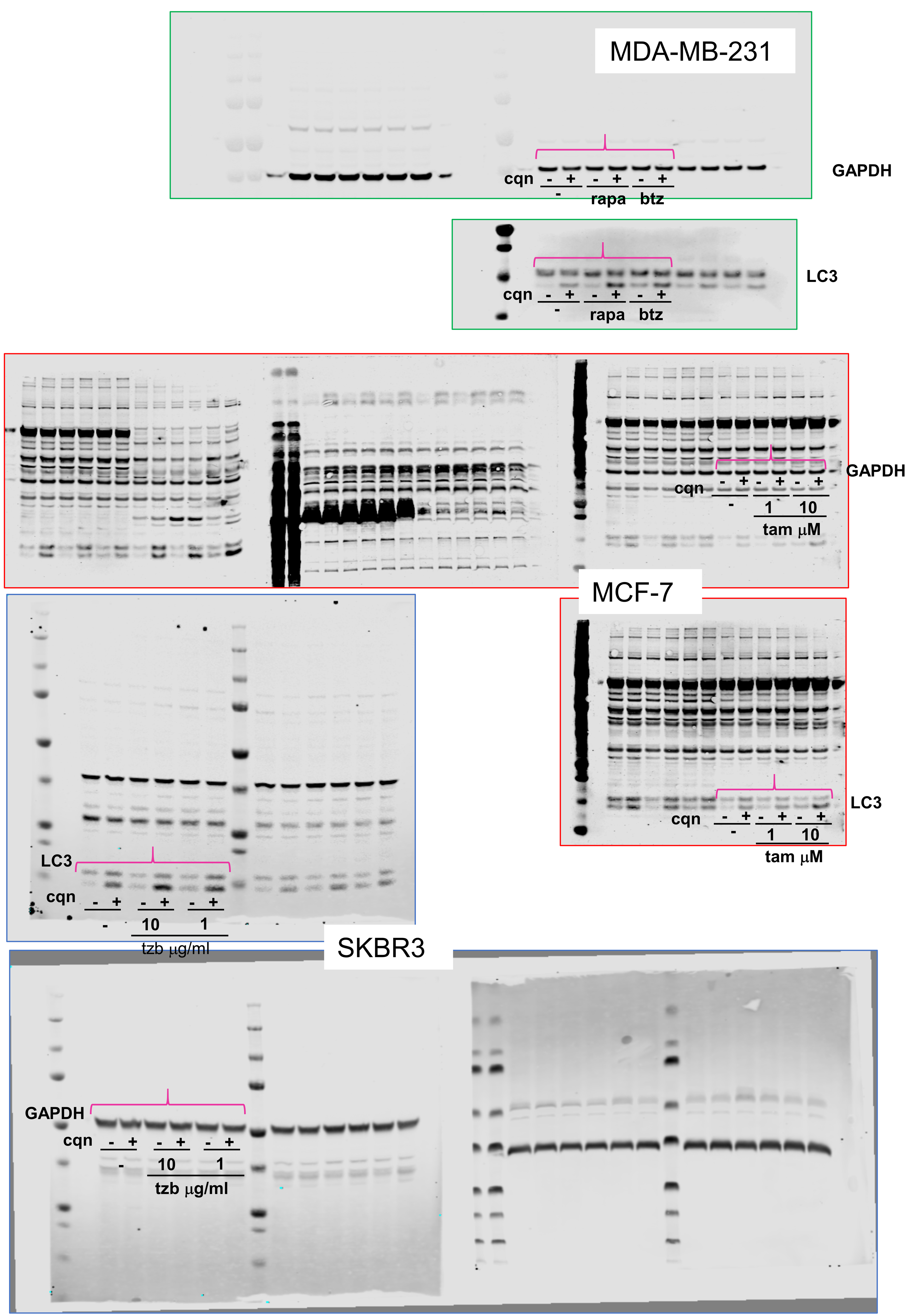

Supplement: S1 Raw image — (TIF) [file pone.0262134.s009.tif]
