# Supplementary material for: Correction: In search of autophagy biomarkers in breast cancer: Receptor status and drug agnostic transcriptional changes during autophagy flux in cell lines
Source: PLoS One. 2025 Jan 15;20(1):e0317835. doi: 10.1371/journal.pone.0317835 (PMC11734928; doi:10.1371/journal.pone.0317835)
Supplement: S1 File — (PDF) [file pone.0317835.s001.pdf]

PLOS ONE

RESEARCH ARTICLE

In search of autophagy biomarkers in breast cancer: Receptor status and drug agnostic transcriptional changes during autophagy flux in cell lines

Francesca Mascia[1], Ilya Mazo[2], Wei-Lun Alterovitz[2], Konstantinos Karagiannis[2], Wells W. Wu[3], Rong-Fong Shen[3], Julia A. Beaver[4], V. Ashutosh Rao[1]*

1 Laboratory of Applied Biochemistry, Division of Biotechnology Review and Research III, Office of Biotechnology Products, CDER, FDA, Silver Spring, Maryland, United States of America, **2** HIVE Bioinformatics Group, Office of Biostatistics and Epidemiology, CBER, FDA, Silver Spring, Maryland, United States of America, **3** Facility for Biotechnology Resource CBER, FDA, Silver Spring, Maryland, United States of America, **4** Oncology Center of Excellence, FDA, Silver Spring, Maryland, United States of America

* ashutosh.rao@fda.hhs.gov

Check for updates

OPEN ACCESS

Citation: Mascia F, Mazo I, Alterovitz W-L, Karagiannis K, Wu WW, Shen R-F, et al. (2022) In search of autophagy biomarkers in breast cancer: Receptor status and drug agnostic transcriptional changes during autophagy flux in cell lines. PLoS ONE 17(1): e0262134. https://doi.org/10.1371/journal.pone.0262134

Editor: Vladimir Trajkovic, Faculty of Medicine, University of Belgrade, SERBIA

Received: June 14, 2021

Accepted: December 18, 2021

Published: January 6, 2022

Copyright: This is an open access article, free of all copyright, and may be freely reproduced, distributed, transmitted, modified, built upon, or otherwise used by anyone for any lawful purpose. The work is made available under the Creative Commons CC0 public domain dedication.

Data Availability Statement: ***PA/E @ ACCEPT: Please check if accession number is functional*** All fastq data files are available on https://www.ncbi.nlm.nih.gov/sra/ with PRJNA732385 as BioProject accession number.

Funding: The authors would like to thank Dr. Thomas Biel (FDA) for critically evaluating the manuscript. This project was supported by an appointment to the Research Participation Program at the Office of Biotechnology Products in

Abstract

Autophagy drives drug resistance and drug-induced cancer cell cytotoxicity. Targeting the autophagy process could greatly improve chemotherapy outcomes. The discovery of specific inhibitors or activators has been hindered by challenges with reliably measuring autophagy levels in a clinical setting. We investigated drug-induced autophagy in breast cancer cell lines with differing ER/PR/Her2 receptor status by exposing them to known but divergent autophagy inducers each with a unique molecular target, tamoxifen, trastuzumab, bortezomib or rapamycin. Differential gene expression analysis from total RNA extracted during the earliest sign of autophagy flux showed both cell- and drug-specific changes. We analyzed the list of differentially expressed genes to find a common, cell- and drug-agnostic autophagy signature. Twelve mRNAs were significantly modulated by all the drugs and 11 were orthogonally verified with Q-RT-PCR (*Klhl24*, *Hbp1*, *Crebrf*, *Ypel2*, *Fbxo32*, *Gdf15*, *Cdc25a*, *Ddit4*, *Psat1*, *Cd22*, *Ypel3*). The drug agnostic mRNA signature was similarly induced by a mitochondrially targeted agent, MitoQ. *In-silico* analysis on the KM-plotter cancer database showed that the levels of these mRNAs are detectable in human samples and associated with breast cancer prognosis outcomes of Relapse-Free Survival in all patients (RSF), Overall Survival in all patients (OS), and Relapse-Free Survival in ER^+ Patients (RSF ER^+). High levels of *Klhl24*, *Hbp1*, *Crebrf*, *Ypel2*, *CD22 and Ypel3* were correlated with better outcomes, whereas lower levels of *Gdf15*, *Cdc25a*, *Ddit4* and *Psat1* were associated with better prognosis in breast cancer patients. This gene signature uncovers candidate autophagy biomarkers that could be tested during preclinical and clinical studies to monitor the autophagy process.

the Center for Drug Evaluation and Research at the U.S. Food and Drug Administration (FDA), administered by the Oak Ridge Institute for Science and Education through an interagency agreement between the U.S. Department of Energy and FDA. The funding source had no part in the study design, data collection/analysis, decision to publish, or preparation of the manuscript.

Competing interests: The authors have declared that no competing interests exist.

Abbreviations: ER, estrogen receptor; PR, progesterone receptor; Her2, human epidermal growth factor receptor 2; Q-RT-PCR, quantitative real time PCR.

Introduction

Autophagy is a highly regulated process of turnover and recycling of damaged cellular components that contributes to maintain cellular homeostasis [1]. The role of autophagy in modulating survival- or death-inducing responses in cancer therapy is debated and both approaches to promote or inhibit autophagy are under intense pre-clinical and clinical investigation [2, 3]. Historically, autophagy has been characterized as a regulator in cancer therapy resistance and autophagy modulators, such as the lysosome inhibitors chloroquine and hydroxychloroquine, are being studied as monotherapy or in combination with other therapies in several clinical trials [2, 4]. Lysosome inhibitors are not specific autophagy inhibitors but are extremely useful tools to characterize autophagy dynamics, such as quantifying the intensity of the autophagy flux *in-vitro* and in some preclinical models [5]. Autophagy flux is an index of autophagic activity that gives a dynamic picture on the intensity of the process induced specifically by various stimuli rather than normal catabolic turnover of cellular components. So far, quantitative measurements of the autophagy flux *in-vivo* have been reported in transgenic animals where the accumulation of fluorescent reporters were used to assess the autophagy process [5]. Clinical studies, however, are limited by lack of robust biomarkers that can be used to monitor and quantify autophagy dynamics *in-vivo*.

We investigated drug-induced autophagy in breast cancer cell lines with differing ER/PR/Her2 status by exposing them to tamoxifen (MCF-7), trastuzumab (SKBR-3), bortezomib and rapamycin (MDA-MB-231) in presence and absence of chloroquine to capture early signs of autophagy flux. Tamoxifen is a non-steroidal selective estrogen receptor (ER) modulator used to treat patients with ER positive breast cancers. Trastuzumab is a recombinant monoclonal antibody that binds to HER2 (human epidermal growth factor receptor 2) and used to treat patients with $HER2^{+}$ breast cancer. Bortezomib is a dipeptide boronic acid derivative and proteasome inhibitor used in patients with multiple myeloma and studied in breast cancer clinical trials. Similarly, rapamycin, is a macrolide compound obtained from *Streptomyces hygroscopicus* that acts as an immunosuppressant. The selected drugs are well known inducers of autophagy, have a resistance mechanism associated with autophagy and are currently used in the clinic either for a specific sub-type of breast cancer or being studied in current clinical trials [2–4]. Even though the above mentioned studies highlight a role for autophagy in modulating various outcomes, each drug activates the autophagy process by its specific mechanism of action. We hypothesized that identifying a common transcriptomic signature, agnostic of drug and receptor status, might offer a clinically relevant autophagy signature that can be used independently of the specific treatment and cell type. While the purpose of this study was not to identify new genes involved in autophagy transcriptional regulation [6], our aim was to identify mRNAs that are actively modulated while the autophagy process is occurring in order to support their potential role as pharmacodynamic markers of autophagy activity during treatment. One could hypothesize that a clinically relevant autophagy signature is one that consistently and specifically reflects gene expression changes during autophagy by multiple drug types and is expressed in the patient target population. We verified this by testing the signature in an independent cohort of samples isolated from relevant cells undergoing autophagy and by performing in silico survival analysis using KM-Plotter. KM-Plotter is an online platform (https://kmplot.com/analysis/) where data collected in publicly available repositories like GEO (Gene expression omnibus) and TCGA (The Cancer Genome Atlas) allow meta-analysis-based discovery and validation of survival biomarkers for several cancer types [7, 8].

Collectively, we present novel findings of an 11-gene signature that was independent of receptor and drug-treatment status and correlated with breast cancer outcomes reported in KM-Plotter and/ or has been previously associated with autophagy processes.

Materials and methods

Cell culture

All cells were purchased from ATCC and regularly tested with Mycoplasma kit (ATCC 30-1012K). MDA-MB-231 cells (ATCC HTB-26) were cultured as reported in [9] while MCF-7 (ATCC HTB-22) and SKBR-3 (ATCC HTB-30) were cultured as suggested in the ATCC catalogue. Cell were cultured for 2 days in 6 well dishes (protein extracts) or 12 well dishes (RNA extracts) before being stimulated with the drugs for the length of time and dose indicated in the figure legends. Rapamycin (R0395), bortezomib (5043140001), tamoxifen (1643306) and chloroquine (C6628) were purchased from Sigma, trastuzumab (Genentech) was purchased from McKesson Corporation. Mitoquinone (MitoQ) was kindly provided by Drs. Joy Joseph and Balaraman Kalyanaraman at the Medical College of Wisconsin (Milwaukee, WI).

Immunoblotting

Proteins were extracted, and lysates were analyzed as previously described in [9]. The GAPDH antibody IMG 5143A was purchased from Imgenex.

RNA analysis

After cell stimulation total RNA was extracted with Trizol and further purified with Qiagen RNAeasy columns. Quality control and library preparation for RNA sequencing were performed as described in [10]. Paired-end sequencing (100 × 2 cycles) of multiplexed mRNA samples was carried out on an Illumina HiSeq 2500 sequencer. Fastq files obtained from the sequencer were generated using bcl2fastq v2.17 (available on https://www.ncbi.nlm.nih.gov/sra/ with **PRJNA732385 as BioProject accession number)** and aligned to the reference human transcriptome [https://www.ncbi.nlm.nih.gov/genome/51 Homo sapiens (human) Reference genome: Homo sapiens (assembly GRCh38.p13) for transcript data, file GCF_000001405.37_GRCh38.p11_rna.fna] using the read aligner Hexagon (v 1.5.1) [11], implemented as a native built-in tool in the FDA HIVE genomics compute infrastructure [12]. Gene-level features were quantified by the Alignment Comparator tool of the FDA HIVE and the output was saved as both the read count file and normalized RPKM file for each sample alignment. The read count files were further processed with DESeq2 [13], version 1.14.1 in R to perform the comparison of the sample groups with p-value < 0.05 (Benjamini-Hochberg corrected for multiple comparison). Four comparisons between untreated samples and drug treated samples were compared and 12 common differentially expressed genes ($p \leq 0.05$ and $-0.5 \leq \log_2 FC \leq +0.5$. FC = fold change) were identified.

Gene-set Variation Analysis (GSVA)

The gene expression matrix of RNASeq hits for all samples was normalized using the count normalization function of the DESeq2 package in R to account for the different read coverage and RNA composition ensuring that a few highly differentially expressed genes do not skew the normalization. Pathway activation was calculated by the tool GSVA [14] in R, using pathway definitions from Molecular Signatures Database (MSigDB) Hallmark gene sets collection developed by Broad Institute (*http://www.gsea-msigdb.org/gsea/msigdb/collections.jsp*). GSVA calculates relative enrichment of a gene set in each sample across the sample space, allowing for sample-wise comparison of gene set enrichment across a dataset. A positive enrichment value for a sample indicates overall higher expression of the genes in the pathway in the sample, compared to the other samples analyzed. The RNASeq hit count data for all treated and untreated breast cancer cell lines, 7 combination, 3 replicas for each, 21 samples in total, were

combined and normalized using DESeq2 package in R prior to submitting to GSVA. Heatmap visualization and clustering of the resulting GSVA score matrix were generated using the heatmap.2 package in R Studio. Clustering used the heatmap.2 default parameters, the complete linkage method and Euclidian distance as a distance metric.

Gene set enrichment analysis

Gene set enrichment analysis (GSEA) was performed on each of the four conditions separately (rapamycin versus untreated, bortezomib versus untreated, tamoxifen versus untreated and trastuzumab versus untreated) using the R package clusterProfiler [15] (version 3.14.3). For each condition the list of genes was sorted based on the shrunk log2 fold generated by the DESeq2 [13] (version 1.26.0) and provided as input for the GSEA [16, 17] using the Molecular Signatures Database (MSigDB) Hallmark gene sets collection developed by Broad Institute (*http://www.gsea-msigdb.org/gsea/msigdb/collections.jsp*) through the msigdbr R package (version 7.2.1) or KEGG pathway [18]. Only sets with the False Discovery Rate corrected p value < 0.05 were considered. For the GSEA analysis 1000 permutations were performed and only sets with more than 10 and less than 500 genes were considered. Enrichment of the custom gene set of the signature 11 genes was assessed by performing an additional GSEA analysis after adding the set of signature genes to the MSigDB. Venn diagrams were generated using the R package VennDiagram (version 1.6.20) to depict the intersection of underrepresented or overrepresented (or both) hallmark sets between different treatments.

Quantitative real time PCR

(Q-RT-PCR): RNA sequencing data were verified on the same samples with real-time quantitative polymerase chain reaction using Power SYBR Green Mix (Thermo Fisher Scientific) and Qiagen QuantiTect Real Time optimized primer assays. RNA was reverse transcribed using the High Capacity cDNA reverse transcription kit (Thermo Fisher Scientific). Q-RT-PCR was performed on a QuantStudio 6 Flex (Thermo Fisher Scientific) using the conditions recommended by the manufacturer. Data are plotted as fold change over basal level with relative expression obtained with Delta Ct method normalized with GAPDH as housekeeping gene. For Q-RT-PCR data statistical differences were tested with unpaired t test using GraphPad Prism software. Statistical significance was identified when $p \leq 0.05$, and higher p values were indicated as "NS" in the graphs.

In silico survival analysis

The association of each of the 12 candidate genes with the outcome in breast cancer datasets was studied in the KM-Plotter (kmplotter.org) online platform [7, 8, 19]. In KM-Plotter, survival was estimated with the Kaplan-Meier method and the Logrank test is used as statistical inference between the two risk groups and the Cox Proportional-Hazards Regression for Survival Data was used to estimate Hazard Ratios. The entire dataset was split in two groups by the median of expression of the gene to be evaluated and a $P < 0.05$ was considered statistically significant. The criteria for outcome were: RFS (relapse-free survival at 5 years), OS (overall survival) and RFS for ER positive patients only. Datasets used for RFS on all cancer samples available or on ER positive cancers only: E-MTAB-365, GSE11121, GSE12093, GSE12276, GSE1456, GSE16391, GSE16446, GSE16716, GSE17705, GSE17907, GSE19615, GSE20271, GSE2034, GSE20685, GSE20711, GSE21653, GSE2603, GSE26971, GSE2990, GSE31519, GSE3494, GSE37946, GSE42568, GSE45255, GSE4611, GSE4922, GSE5327, GSE6532, GSE7390, GSE9195. Datasets used for OS are: GSE1456, GSE16446, GSE16716, GSE20271, GSE20685, GSE20711, GSE3494, GSE37946, GSE42568, GSE45255, GSE7390. In addition to

testing the association of each of the single candidate genes with the outcome in breast cancer datasets, we also tested the association of the average of the 11 gene expression values with the outcome using the same criteria for the outcome as the individual genes (RFS, OS and RFS for ER positive patients only). Phlda gene was excluded from the panel as it did not pass the PCR validation. For the analysis, we selected the "inverted values" for the genes that showed low expression to correlate with better outcome (Gdf15, Ddit4, Cdc25, Psat1), and the "actual (non-inverted) values" for the genes for which the high expression correlates with better outcome (Klhl24, Fbxo32, Crebrf, HBP1, CD22, Ypel2, Ypel3).

Autophagy genes analysis (heatmap and volcano plot)

The list of 232 autophagy-related genes were obtained from the HADb Human Autophagy Database (http://autophagy.lu/clustering/index.html). This autophagy gene list was used as a filter for the normalized count matrix. The gene expression count matrix after filtering was visualized using heatmap.2 package in R. The bioinfokit package in Python was used to visualize the volcano plot for the autophagy gene list using the most conservative 'blended' values from four differential expression results (the larges P-value out of four P-values, and the logFC with the smallest absolute value). The thresholds were set as follows: P-value < 0.05; abs $(\log_2 FC) > 0.2$.

Results

Autophagy flux was triggered in breast cancer cell lines with different receptor status, MCF-7, MDA-MB-231, and SKBR-3 by exposing them to tamoxifen, bortezomib, rapamycin, or trastuzumab, respectively (Fig 1A).

Drug induced autophagy flux was measured by observing the difference in LC3II accumulation in presence and absence of chloroquine by western blot. Cells were either left untreated or stimulated with the above-described drugs in the presence or absence of chloroquine. Chloroquine, by blocking the lysosomal fusion, confirms the accumulation of the lipidated form of the protein LC3, LC3 II, that increases proportionally to the activation of the autophagic flux process [5]. In Fig 1B, western blot for MCF-7 protein extracts showed the activation of the autophagy flux after 16 hours of tamoxifen treatment (1 and 10 μM). Densitometry of the LC3 II bands of the chloroquine treated cell extracts (lane 6 compared to lane 2) normalized to GAPDH levels showed a two-fold increase after drug treatment at the higher dose. In panel C, we report the intensity of the autophagy flux in MDA-MB-231 cells after treatment for 8 hours with rapamycin 200 nM or bortezomib 1μM. LC3 II levels after drug treatments were respectively 1.7x and 1.6x the intensity of the untreated band. Similarly, in panel D, 16 hours of trastuzumab 10 μg/ml showed an increase of the autophagy flux of 1.7x with respect to the basal level.

We collected total RNA under the cell culture conditions above when the cells were in the early phase of the drug induced autophagy response. For each drug and cell line three replicates for untreated or drug treated RNA samples (red marked samples in Fig 1B–1D) were isolated and sequenced to first generate a drug-specific gene expression profile. A Venn diagram with each of the differentially expressed genes from the four groups of drug treatments is depicted in Fig 1E. Only twelve mRNAs were in common between the differentially expressed genes (adjusted $p \leq 0.05$ and $-0.5 \leq \log_2 FC \leq +0.5$, FC = fold change) during the autophagy flux induced by all 4 drugs and are collectively referred to as the drug-independent cell-independent autophagy signature mRNAs. The full sequencing data for each of the 12 common signature mRNAs are described in Fig 2. Only mRNAs that were upregulated or downregulated significantly by the drugs in all the cells were selected for further analyses.

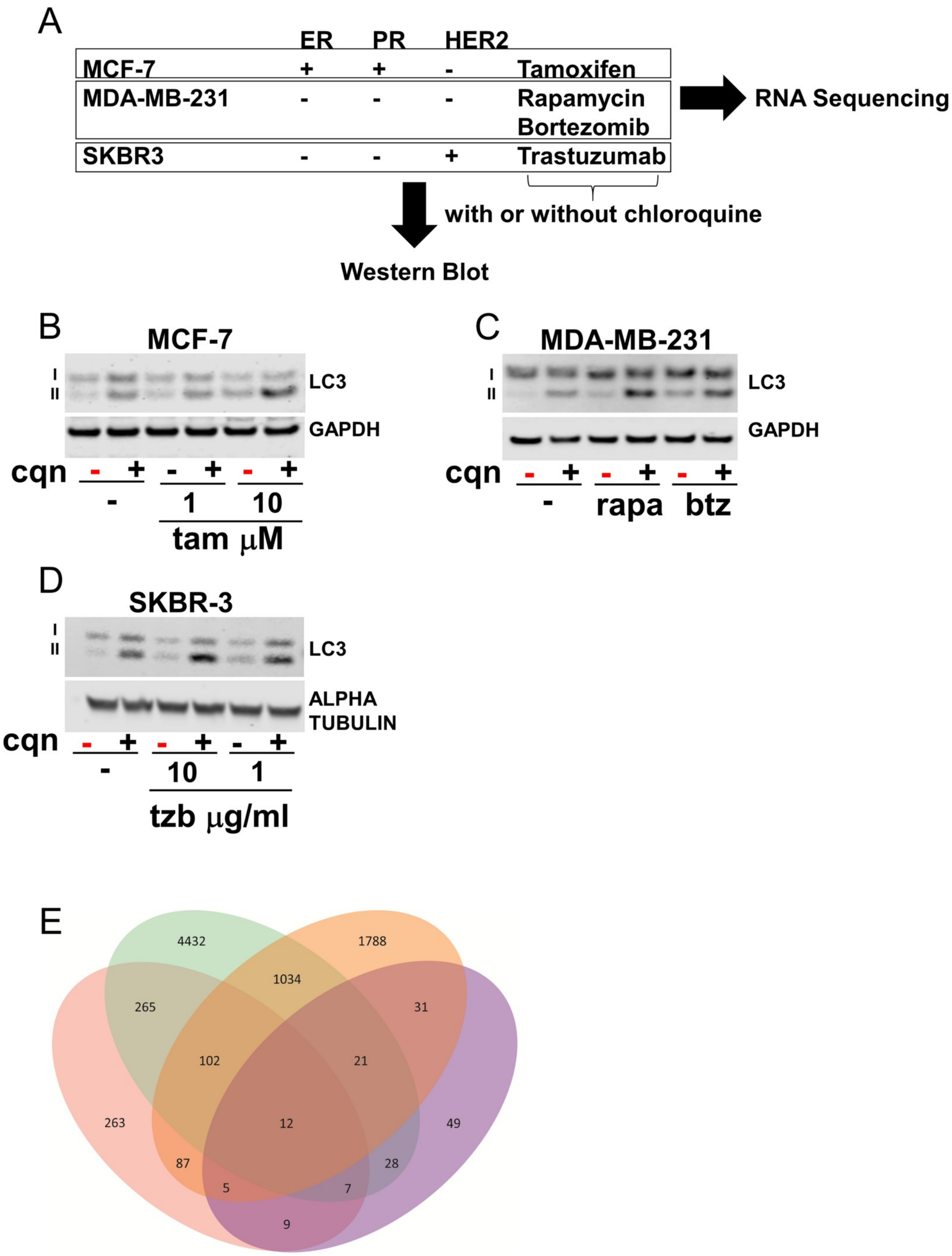

Fig 1. Drug induced autophagy flux and transcriptional profiling in breast cancer cell lines. A) Schematic with receptor status, cell lines and drugs used for western blot and sequencing analysis. B) MCF-7 cells treated with tamoxifen (tam) 1 and 10 μM in presence and absence of chloroquine (cqn) 10 μM for 16 hours. C) MDA-MB-231 cells treated with rapamycin (rapa) 200 nM and bortezomib (btz) 1 μM in presence and absence of chloroquine 10 μM for 8 hours. D) SKBR-3 cells treated with trastuzumab (tzb) 10 and 1 μg/ml in presence and absence of chloroquine 10 μM for 16 hours. Samples marked with red represent the conditions chosen for the RNA extraction for sequencing experiments. E) Venn diagram with common genes between the 4 groups of treatments. Each color represents the unique subset of genes from the differential expression analysis between no treatment and drug treatment (pink = rapamycin 200 nM, green = bortezomib 1 μM, orange = tamoxifen 10 μM, purple = trastuzumab 10 μg/ml).

https://doi.org/10.1371/journal.pone.0262134.g001

Transcriptional expression is depicted as fold changes over the basal levels for all the common mRNAs in MDA-MB-231, MCF-7 and SKBR-3 cells. All tested drugs upregulated levels

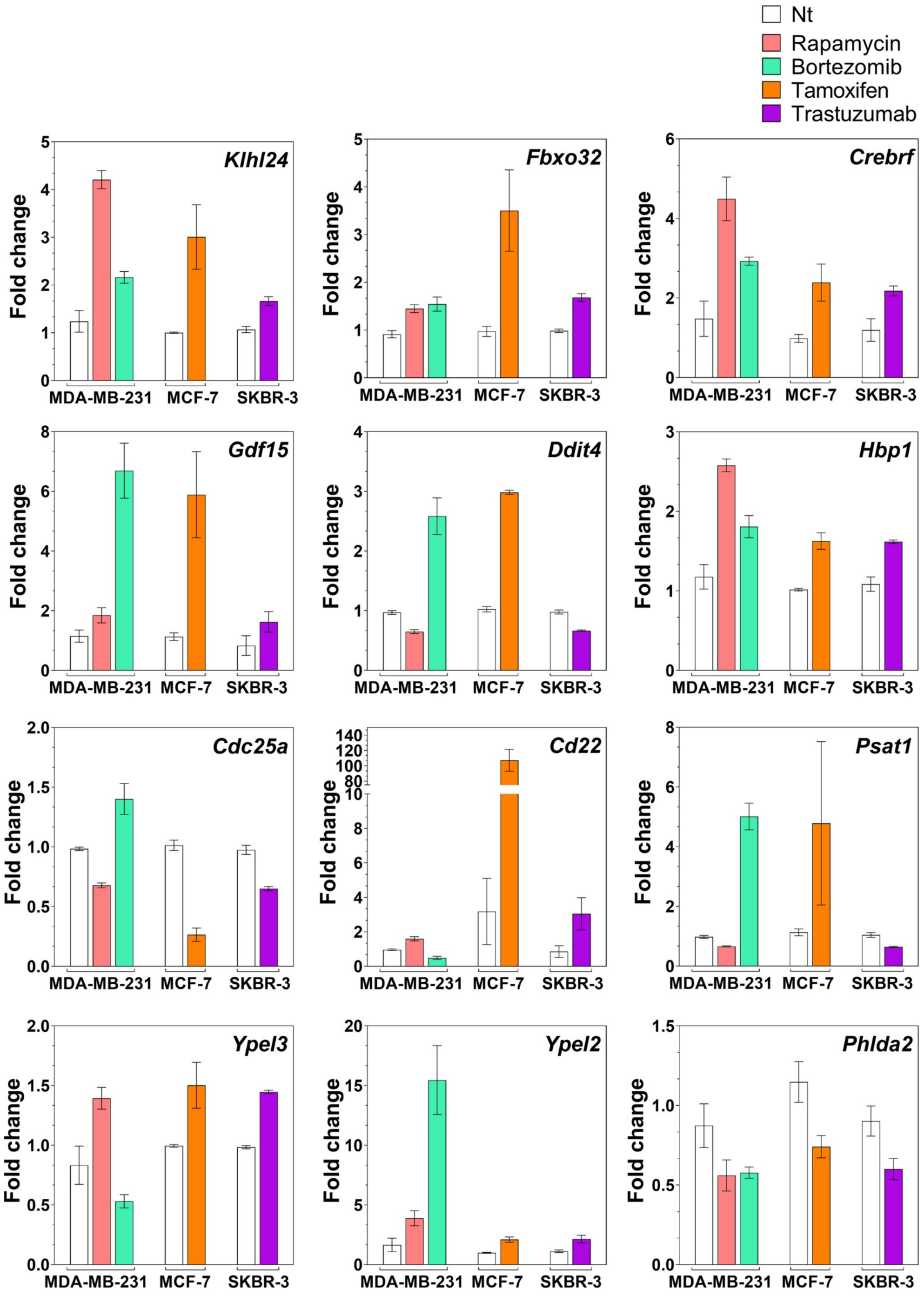

Fig 2. RNA sequencing data on the drug-independent cell-independent signature. Relative expression of the 12 mRNAs plotted as fold change over the non-treated for each drug and cell line. The mRNAs were selected from the lists of differentially expressed genes between no treatment and drug treatment in each group with $p \leq 0.05$ and $-0.5 \leq \log_2 FC \leq +0.5$. FC = fold change.

https://doi.org/10.1371/journal.pone.0262134.g002

of *Klhl24*, *Fbxo32*, *Crebrf*, *Gdf15*, *Hbp1* and *Ypel2*. Bortezomib and tamoxifen upregulated *Ddit4 and Psat1* mRNA levels while rapamycin and trastuzumab downregulated the levels of those mRNAs. For *Cd25a* and *Cd22*, bortezomib, tamoxifen and trastuzumab respectively

downregulated and upregulated the mRNA levels while rapamycin had the opposite effect on both. Finally, *Phlda2* mRNA levels that were downregulated by all drugs treatment were not verified using Q-RT-PCR as with the other mRNAs identified by RNA sequencing (S1 and S2 Figs). Hence, we excluded *Phlda2* from the overall signature reported.

Gene set variation analysis (GSVA) provides an estimate of pathway activity by transforming an input gene-by-sample expression data matrix into a corresponding gene-*set*-by-sample expression data matrix (i.e. a pathway expression score matrix). The clustering of the GSVA pathway scores showed high reproducibility of the data as the triplicates clustered together. In Fig 3 the heatmap diagram shows the gene set variation analysis between the samples obtained from the sequencing experiment.

We performed gene set enrichment analysis (GSEA) using the hallmark gene sets. S3A Fig showed that "E2F targets" and "G2M checkpoint" positively correlated with all treatments while the gene set "TNFα signaling via NF-κB" negatively correlate with all treatments. Furthermore, hallmark gene sets "MYC targets V2" and "MYC targets V1" positively correlated with rapamycin, tamoxifen and trastuzumab but negatively correlate with bortezomib. Finally, "unfolded protein response" and "MTORC1 signaling" hallmark sets showed positive correlation with rapamycin and trastuzumab and negative correlation with tamoxifen and bortezomib. Using this information, we generated Venn diagrams where the intersection depicted the underrepresented Hallmark sets between different treatments (S3B Fig). Only one set, "TNFA signaling via NF-κB", was found to be underrepresented in cells of all four treatments when compared to control. A second Venn diagram describes the intersection of the overrepresented hallmark sets between the different treatments (S3C Fig). Only two sets "E2F targets" and "G2M checkpoint", were found to be overrepresented in cells of all four treatments when compared to control. Finally, seven gene sets were found common among all treatments when considering Hallmark gene sets significantly correlated to each treatment but irrespective of positive or negative correlation (S3D Fig and S1 Table). When gene set enrichment was performed using KEGG pathways (S4 Fig), the analysis showed that the four different treatments impacted different pathways with Tamoxifen treatment altering the smallest number of pathways.

Next, we specifically queried if autophagy-related genes were transcriptionally modulated during the autophagy flux induced by cancer therapy treatments (S5A Fig). We obtained the list of literature-curated 232 autophagy-related genes from the HADb (Human Autophagy Database http://autophagy.lu/clustering/index.html) and used it as a filter for the normalized count matrix visualized as a heatmap. Shown in S5 Fig, several autophagy-related genes were modulated by at least one drug treatments but not all of the drugs at the same time, so they do not fit our drug-independent transcriptomic signature criteria. The volcano plot of the expression levels of the HADb gene list (S5B Fig) highlighted that only *Klhl24* is differentially expressed with a $\log_2$ fold chance between -0.5 and 0.5. The other 10 signature mRNAs have not been previously associated with autophagy.

Mitoquinone is a mitochondrially-targeted agent and a known potent inducer of selective autophagy in MDA-MB-231 cells [20]. In Fig 4A, we report the mRNA levels of the autophagy signature mRNAs as a fold change over the corresponding non-treated sample after 5 μM mitoquinone exposure for 18 hours.

All eleven mRNAs verified with Q-RT-PCR showed significant changes with mitoquinone treatment while *Phlda2* mRNA (the mRNA that did not pass the Q-RT-PCR confirmation) did not change significantly. While all the mRNAs were upregulated during autophagy flux, only *Cdc25a* was downregulated. Thus, the 11 mRNAs signature confirms mRNAs that are differentially modulated during autophagy induction in a new cohort of samples using an orthogonal and unrelated autophagy inducer.

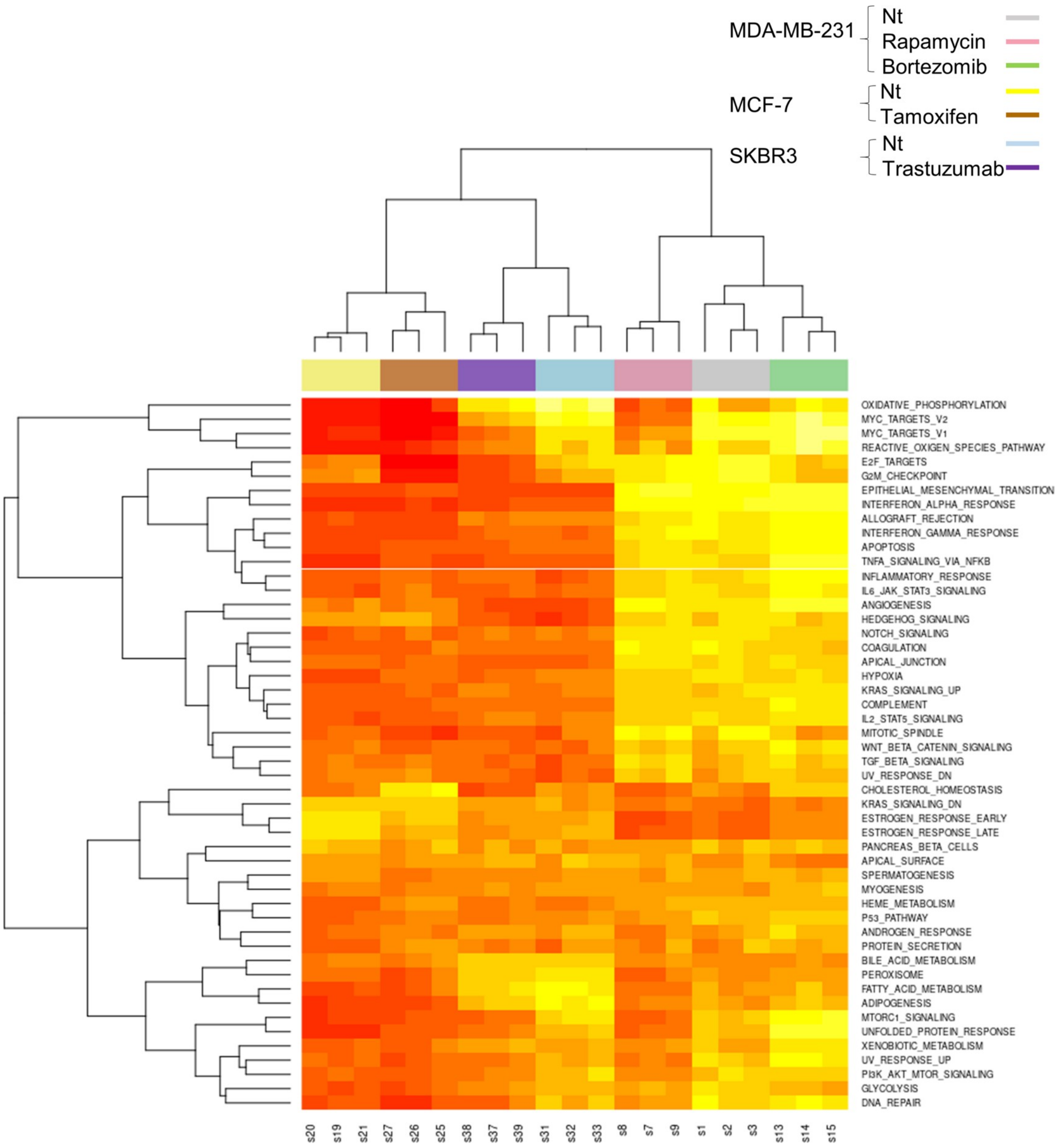

Fig 3. Drug induced gene set variation analysis and differential gene expression hierarchical clustering heatmap of gene set variation analysis enrichment scores of hallmark pathways from RNASeq data. These data included n = 3 replicates that are clustered under colored bar according to the treatment and cell line.

https://doi.org/10.1371/journal.pone.0262134.g003

To verify if the mRNAs of the drug-independent-cell independent autophagy signature are expressed *in vivo* in breast cancer patients and how they correlate with clinical outcome measures, we queried the KMplotter database (kmplotter.org) [7, 8, 19]. The levels of expression of 10 out of the 11 mRNAs were significantly correlated with at least one reported patient outcome (Fig 4B). In this table we summarize the level of expression of the signature mRNAs in patients (H for high and L for low) and correlation with reported outcomes of Relapse-Free Survival in all patients (RSF), Overall Survival in all patients (OS), and Relapse-Free Survival in ER^+ Patients (RSF ER^+). Detailed data for every mRNA and each correlated outcome are depicted in S6–S8 Figs.

Not all signature mRNA levels were correlated with all three outcomes. In Fig 4B we included the highest *p* value for each significant outcome association. If more than one

A

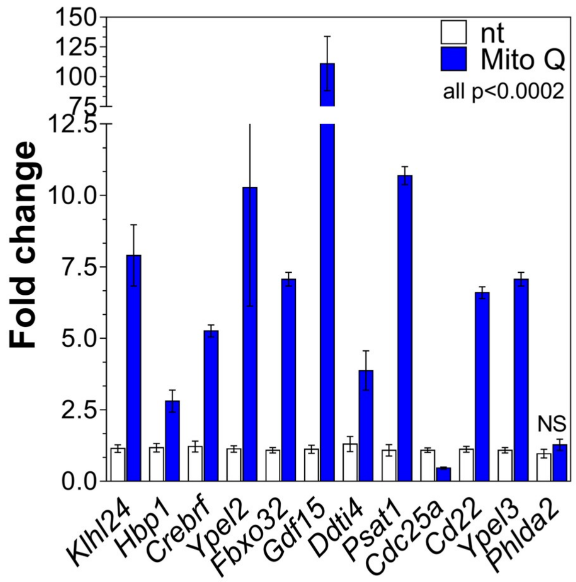

B

	Pearson r RT/NGS	Trend *in vitro*	Patient prognosis	p value KM plotter analysis	Number of publications
Klhl24	**0.96**	↑	**H**	**p≤ 6.5e-07 (RFS/RFS ER⁺)**	**H A D / pubmed 1**
Hbp1	**0.93**	↑	**H**	**p≤ 2.2e⁻⁰⁵ (RFS/OS/RFS ER⁺)**	**pubmed 1**
Crebrf	**0.90**	↑	**H**	**p≤ 0.00021 (RFS/OS/RFS ER⁺)**	**pubmed 4**
Ypel2	**0.99**	↑	**H**	**p≤ 0.0031 (RFS/OS/RFS ER⁺)**	**pubmed 0**
Fbxo32	**0.90**	↑	**NS**	**NS**	**pubmed 58**
Gdf15	**0.94**	↑	**L**	**p≤ 0.016(RFS ER⁺/OS)**	**pubmed 7**
Cdc25a	**0.88**	↓	**L**	**p≤ 5e⁻⁰⁴ (RFS/OS/RFS ER⁺)**	**pubmed 9**
Ddit4	**0.97**	↑↓	**L**	**p≤ 0.0031 (RFS/OS/RFS ER⁺)**	**KEGG / pubmed 51**
Psat1	**0.85**	↑↓	**L**	**p≤ 0.009 (RFS/OS)**	**pubmed 2**
Cd22	**0.93**	↑↓	**H**	**p≤ 0.0038 (OS)**	**pubmed 0**
Ypel3	**0.63**	↑	**H**	**p≤ 0.0059 (RFS/OS/RFS ER⁺)**	**pubmed 1**

C

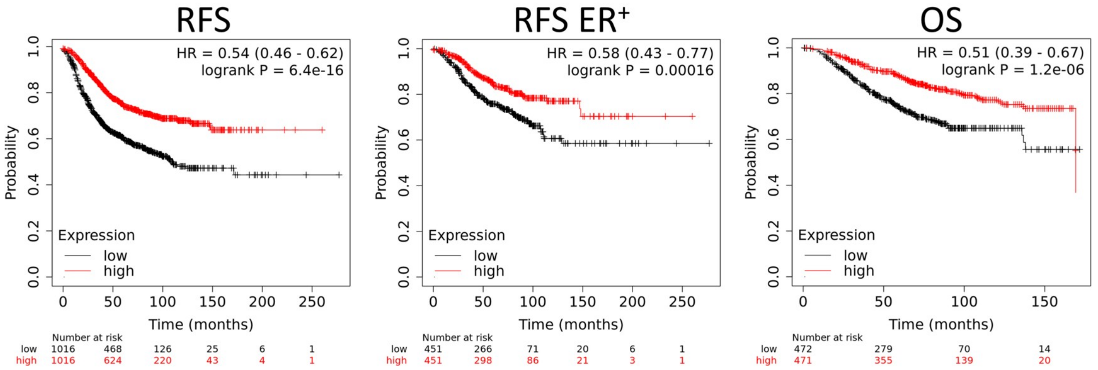

Fig 4. Testing the autophagy mRNA signature in an independent cohort of mRNAs in vitro and in a database of samples from breast cancer patients. A) MDA-MB-231 cells were treated with 5 μM mitoquinone (Mito Q), a well characterized inducer of autophagy, for 16 hours. Relative expression levels of the mRNAs from the autophagy signature were verified by Q-RT-PCR and depicted in the graph as fold change upon untreated levels. B) The table lists: Pearson r value of correlation between RNA Q-RT-PCR data and sequencing data; trend of the expression of the mRNA after the drug treatments where ↑ means upregulation, ↓ means downregulation and ↑↓ means upregulated or downregulated depending on the drug/cell line; correlation with better patient prognosis in different outcomes such as relapse-free survival (RFS), overall survival (OS), relapse-free survival in ER^+ patients only (RFS ER^+) with high levels of mRNA expression (H) or low levels of mRNA expression (L); NS = non-significant $p \geq 0.05$; Higher p value for the outcome correlations described in the previous column; Number of publications on PubMed with the term search "autophagy and mRNA gene symbol" as of April 2021 and the presence of the mRNA in the human autophagy database (HAD) or in the Kyoto Encyclopedia of Genes and Genomes (KEGG) as associated with an

autophagy pathway. C) Cox Proportional Hazards Regression for the group of 11 mRNAs. High (red line) or low (black line) levels of the average 11 gene expression values are correlated with patient's outcomes with KM Plotter software. Patients' outcomes studied are: RFS (relapse-free survival), OS (overall survival) and RFS for ER positive patients only. P values are in the top left of each graph.

https://doi.org/10.1371/journal.pone.0262134.g004

outcome is indicated in the parenthesis, the *p* value for each of those outcomes were lower or equal to the indicated value. High levels of the mRNAs for *Klhl24*, *Hbp1*, *Crebrf*, *Ypel2*, *CD22 and Ypel3* were correlated with better outcomes. In contrast, lower levels of the mRNAs for *Gdf15*, *Cdc25a*, *Ddit4* and *Psat1* were associated with better prognosis in breast cancer patients. In this broad analysis, *Fbxo32* mRNA levels were not correlated with patient outcome.

When we analyzed all the expression levels of the 11 mRNAs as a group, we found even a stronger association with RSF ($p = 6.4x10^{-16}$), RFS ER^+ ($p = 0.00016$) and OS ($p = 1.2x10^{-6}$) (Fig 4C).

Finally, we surveyed PubMed with the search terms [mRNA name] and [autophagy] to identify if the genes in the signature list were previously associated with the autophagy process (Fig 4B). As of April 2021, we found that *Fbxo32* was present in 58 publications. Additionally, we examined the human autophagy database (HADb http://autophagy.lu/) and found only one of the signature mRNAs, *Klhl24*. Finally, the Kyoto Encyclopedia of Genes and Genomes (KEGG https://www.genome.jp/kegg/pathway.html), reports *Ddit4* in the autophagy pathway. Thus, nine mRNAs have been previously associated with autophagy in the literature while Ypel2 and Cd22 appear novel and worthy of further investigation.

We consider each of these transcriptomic findings to be novel, hypothesis-generating, and warranting additional studies in the context of autophagy and breast cancer treatment.

Discussion

Autophagy is a fundamental mechanism that is conserved from yeast to mammals and has been mostly characterized by genetic approaches and using non-human test models [1, 5]. A major limitation of available autophagy inhibitors used in human studies is their lack of specificity combined with a lack of specific and quantitative biomarkers that could be leveraged for targeting autophagy in human diseases. For instance, a specific and quantitative measurement of autophagy *in-vivo* is currently limited to the count of autophagosomes by transmission electron microscopy (TEM) which is not a practical approach in a clinical setting [5].

Since autophagy is a common survival response after cancer treatment, we speculated that although acting through different mechanisms, the autophagy inducing drugs could have a common transcriptional program that is occurring during an active autophagy flux. We examined mRNA as the most sensitive method for quantitation and rapid clinical applications. Autophagy is both a post-transcriptional and a transcriptional mechanism and its master regulators have been previously reviewed [1, 6]. Transcriptional signatures that highlight the importance of autophagy genes, their genetic variants or their related non-coding RNAs have been demonstrated to predict prognosis in breast cancer [21–24]. Our aim was to find specific mRNAs that are transcriptionally upregulated or downregulated during the autophagy process that provides a specific and quantitative measure of the process itself. We used several known autophagy inducers and provide orthogonal measurement of the transcriptional profile by RNA sequencing and Q-RT-PCR. While several drug specific pathways were activated (Fig 1E), only 12 common autophagy-related mRNAs were differentially expressed with 11 of them and verified with Q-RT-PCR (S1 Fig). Based on PubMed, some (*Ypel2* and *Cd22*) have not been previously associated with autophagy and represent novel leads into autophagy signaling.

A search for [Klh24] and [autophagy] retrieved only 1 publication even though the association between autophagy and *Klhl24* is present in the human autophagy database (HADb). Indeed, *Klhl24* is a validated target of an established autophagy related microRNA (miR 124) [25], and one of the mRNAs modulated by autophagy-inducing stimuli like rapamycin and serum starvation in cancer cells [26]. In our volcano plot depiction of the autophagy associated genes, *Klhl24* emerged as the only gene that changed significantly during the autophagy flux (S5B Fig). Most of the genes previously associated with autophagy regulate this process at the protein level. During drug induced autophagy flux, the levels of autophagy associated genes mRNA did not change significantly in all drug induced conditions. Thus, their mRNA levels were not optimal to reliably measure changes in autophagy flux.

We summarized several observations on every mRNA of the signature in Fig 4B where we describe if a correlation with better prognosis in patient outcomes is associated with higher or lower values of expression of the mRNA. In the case of *Klhl24*, a better probability of relapse-free survival in all breast cancer patients (RSF) and ER^+ only patients (RSF ER^+) was associated with higher levels of the mRNA with a $p \leq 6.5X10^{-7}$. Our data suggests that *Klhl24* mRNA can be used to monitor autophagy in patients undergoing breast cancer therapy. Studies that use autophagy inhibitors or activators in combination with cancer therapies, with adequate sampling and follow up with survival outcomes, could benefit from linking the autophagy levels monitored through these mRNAs to the outcome. Indeed, very recently *Klhl24* has been identified as part of an autophagy related signature in acute myeloid leukemia [27]. The only mRNAs from our studies that was not linked to an outcome in breast cancer is *Fbxo32* also called Atrogin1. Interestingly, several publications that describe its role in autophagy primarily related to its role in muscle atrophy. The other mRNA in the signature with abundant autophagy-related publications is *Ddit4* also called *Redd1*. In one study, the authors used KM-plotter and other online tools to evaluate the association of *Ddit4* with several types of malignancies [7]. In this paper, *Ddit4* is described as a prognostic biomarker in several malignancies including breast cancer. Thus, we do not exclude that the mRNAs found in the signature, or their protein counterparts, could have a wider role in cancer outcome and or autophagy regulation but further studies are necessary to clearly distinguish and establish these claims. The mRNA signature uncovers autophagy candidate biomarkers that can be easily measured during preclinical and clinical studies to monitor the autophagy process and can be used to accelerate development of specific autophagy modulators and more effective and targeted cancer therapies.

Supporting information

S1 Fig. Q-RT-PCR verification on the drug-independent cell-independent signature. Relative expression of the 12 mRNAs plotted as fold change over the non-treated for each drug and cell line. All the comparisons between untreated and drug treated within the same cells are significant with $p \leq 0.05$ except when labeled with NS = non-significant ($p > 0.05$).
(TIF)

S2 Fig. Correlation analysis between Q-RT-PCR and RNA sequencing data on the autophagy mRNA signature. Correlation graphs with fold change data from Q-RT-PCR on Y axis and fold change data from the RNA sequencing data (NGS) on X axis. The numbers in the top left corner represent the Pearson r value.
(TIF)

S3 Fig. Gene set enrichment analysis from the drug induced differentially expressed genes. A) GSEA normalized enrichment score for each hallmark gene set in rapamycin, bortezomib,

tamoxifen and trastuzumab treated cells. Positive normalized enrichment scores indicate gene sets that are positively enriched in each treatment compared to control. Gene sets colored in darker blue indicate higher statistical significance. Only sets with FDR < 0.05 are included in the results. B) Venn diagram showing the overlap of significantly underrepresented hallmark gene sets between rapamycin (pink), bortezomib (green), tamoxifen (orange) and trastuzumab (purple) treated cells. C) Venn diagram showing the overlap of significantly overrepresented hallmark gene sets between drug treatments. D) Venn diagram showing the overlap of significantly correlated hallmark gene sets between drug treatments.
(TIF)

S4 Fig. KEGG gene set enrichment analysis from the drug induced differentially expressed genes. GSEA normalized enrichment score for each KEGG pathway in rapamycin, bortezomib, tamoxifen and trastuzumab treated cells. Positive normalized enrichment scores indicate pathways that are positively enriched in each treatment compared to control. Gene sets colored in darker blue indicate higher statistical significance. Only pathways with FDR < 0.05 are included in the results.
(TIF)

S5 Fig. Autophagy associated genes transcriptional changes during autophagy flux. A) Hierarchical clustering heatmap of gene expression values for the list of 232 autophagy-related genes from the Human Autophagy Database in RNASeq data from untreated and treated breast cancer cell lines. The arrow indicates *Klhl24* mRNA. B) Volcano plot for 232 autophagy genes from the Human Autophagy Database. Red and blue colored dots show genes that pass the P-value threshold of <0.05 (horizontal line). The vertical lines are log2 fold change values <-0.5 and >0.5 (red dot).
(TIF)

S6 Fig. Evaluation of autophagy signature on the relapse-free survival of breast cancer patients. High (red line) or low (black line) levels of each gene are correlated with patient's outcome with KM Plotter software. P values are in the top right of each graph.
(TIF)

S7 Fig. Evaluation of autophagy signature on the overall survival of breast cancer patients. High (red line) or low (black line) levels of each gene are correlated with patient‘s outcome with KM Plotter software. P values are in the top right of each graph.
(TIF)

S8 Fig. Evaluation of autophagy signature on the relapse-free survival of ER^+ breast cancer patients. High (red line) or low (black line) levels of each gene are correlated with patient‘s outcome with KM Plotter software. P values are in the top right of each graph.
(TIF)

S1 Raw image. Original uncropped images underlying all western blot membrane scans used in Fig 1.
(TIF)

S1 Table. Logical matrix of significantly over-/under-represented or altered (over-represented in some conditions and under-represented in others) gene sets in cells treated with rapamycin, bortezomib, tamoxifen and trastuzumab. Gene sets observed to be significantly over-/under-represented or altered in all conditions are highlighted in yellow. Note that the set of altered gene sets is a super-set of over-represented and under-represented gene sets.
(XLSX)

Acknowledgments

The authors would like to thank Dr. Thomas Biel and Elliot Rosen (FDA) for critically evaluating the manuscript and for assistance with electronic files. The views expressed in this article are those of the authors and do not necessarily reflect the official policy or position of the United States Food and Drug Administration and the Department of Health and Human Services, nor does mention of trade names, commercial products, or organizations imply endorsement by the United States Government.

Author Contributions

Conceptualization: Francesca Mascia, Julia A. Beaver, V. Ashutosh Rao.

Data curation: Ilya Mazo, Wei-Lun Alterovitz, Konstantinos Karagiannis, Wells W. Wu, V. Ashutosh Rao.

Formal analysis: Francesca Mascia, Ilya Mazo, Wei-Lun Alterovitz, Konstantinos Karagiannis, Wells W. Wu, V. Ashutosh Rao.

Funding acquisition: V. Ashutosh Rao.

Investigation: Francesca Mascia, Wells W. Wu, V. Ashutosh Rao.

Methodology: Francesca Mascia, Ilya Mazo, Wei-Lun Alterovitz, Konstantinos Karagiannis, Wells W. Wu, Rong-Fong Shen, V. Ashutosh Rao.

Project administration: Konstantinos Karagiannis, Rong-Fong Shen, V. Ashutosh Rao.

Resources: V. Ashutosh Rao.

Software: Ilya Mazo, Wei-Lun Alterovitz, Konstantinos Karagiannis.

Supervision: Konstantinos Karagiannis, Rong-Fong Shen, V. Ashutosh Rao.

Validation: Francesca Mascia, Ilya Mazo, Konstantinos Karagiannis, Wells W. Wu.

Visualization: Ilya Mazo, Wei-Lun Alterovitz, Konstantinos Karagiannis.

Writing – original draft: Francesca Mascia, Ilya Mazo, Wei-Lun Alterovitz, V. Ashutosh Rao.

Writing – review & editing: Francesca Mascia, Ilya Mazo, Wei-Lun Alterovitz, Konstantinos Karagiannis, Wells W. Wu, Rong-Fong Shen, Julia A. Beaver, V. Ashutosh Rao.

References

1. Bento CF, Renna M, Ghislat G, Puri C, Ashkenazi A, Vicinanza M, et al. Mammalian autophagy: how does it work? Annu Rev Biochem. 2016; 85:685–713. https://doi.org/10.1146/annurev-biochem-060815-014556 PMID: 26865532
2. Mulcahy Levy JM, Towers CG, Thorburn A. Targeting autophagy in cancer. Nature Rev Cancer. 2017; 17:528–542.
3. Fulda S, Kogel D. Cell death by autophagy emerging molecular mechanisms and implication for cancer therapy. Oncogene. 2015; 34:5105–5113. https://doi.org/10.1038/onc.2014.458 PMID: 25619832
4. Kanta Das C, Mandal M, Kogel D. Pro-survival autophagy and cancer cell resistance to therapy. Cancer Metastasis Rev. 2018; 37:749–766. https://doi.org/10.1007/s10555-018-9727-z PMID: 29536228
5. Klionsky DJ, Abdelmohsen K, Abe A, Abedin MA, Abeliovich H, Avecedo Arozena A, et al. Guidelines for the use and interpretation of assays for monitoring autophagy. Autophagy. 2016; 12:1–222. https://doi.org/10.1080/15548627.2015.1100356 PMID: 26799652
6. Fullgrabe J, Ghislat G, Cho DH, Rubinsztein DC. Transcriptional regulation of mammalian autophagy at a glance. J. Cell Science. 2016; 29:3059–3066. https://doi.org/10.1242/jcs.188920 PMID: 27528206

7. Pinto JA, Rolfo C, Raez LE, Prado A, Araujo JM, Bravo L, et al. In silico evaluation of DNA damage inducible transcript 4 gene as prognostic biomarker in several malignancies. Sci Rep. 2017; 7:1526–1536. https://doi.org/10.1038/s41598-017-01207-3 PMID: 28484222

8. Nagy A, Lanczky A, Menyhart O, Gyorffy B. Validation of miRNA prognostic power in hepatocellular carcinoma using expression data of independent datasets. Sci Rep. 2018; 8:9227–9235. https://doi.org/10.1038/s41598-018-27521-y PMID: 29907753

9. Biel TG, Rao VA. Mitochondrial dysfunction activates lysosomal-dependent mitophagy selectively in cancer cells. Oncotarget. 2018; 9:995–1011. https://doi.org/10.18632/oncotarget.23171 PMID: 29416672

10. Wu WW, Phue JN, Lee CT, Lin C, Xu L, Wang R, et al. Robust sub-nanomolar library preparation for high throughput next generation sequencing. BMC Genomics. 2018; 19:326–336. https://doi.org/10.1186/s12864-018-4677-y PMID: 29728062

11. Santana-Quintero L, Dingerdissen H, Thierry-Mieg J, Mazumder R, Simonyan V. HIVE-hexagon: high-performance, parallelized sequence alignement for next-generation sequencing data analysis. PLoS One. 2014; 9:1–11.

12. Simonyan V, Chumakov K, Dingerdissen H, Faison W, Goldweber S, Golikov A, et al. High performance integrated virtual environment (HIVE): a robust infrastructure for next-generation sequence data analysis. Database. 2016; 2016:1–16. https://doi.org/10.1093/database/baw022 PMID: 26989153

13. Love MI, Huber W, Anders S. Moderated estimation of fold change and dispersion for RNA-seq data with DESeq2. Genome Biology. 2014; 15:550–570. https://doi.org/10.1186/s13059-014-0550-8 PMID: 25516281

14. Hänzelmann S, Castelo R, Guinney J. GSVA: Gene set variation analysis for microarray and RNA-seq data. BMC Bioinformatics. 2013; 14:7. https://doi.org/10.1186/1471-2105-14-7 PMID: 23323831

15. Wu T, Hu E, Xu S, Chen M, Guo P, Dai Z, et al. clusterProfiler 4.0: A universal enrichment tool for interpreting omics data. Innovation (N Y). 2021; 3:100141. https://doi.org/10.1016/j.xinn.2021.100141 PMID: 34557778

16. Subramanian A, Tamayo P, Mootha VK, Mukherjee S, Ebert BL, Gillette MA, et al. Gene set enrichment analysis: A knowledge-based approach for interpreting genome-wide expression profiles. PNAS. 2005; 43:15545–15550. https://doi.org/10.1073/pnas.0506580102 PMID: 16199517

17. Mootha V, Lindgren C, Eriksson KF, Subramanian A, Sihag S, Lehar J et al. PGC-1α-responsive genes involved in oxidative phosphorylation are coordinately downregulated in human diabetes. Nat Genet. 2003; 34:267–273. https://doi.org/10.1038/ng1180 PMID: 12808457

18. Kanehisa M, Goto S. KEGG: Kyoto Encyclopedia of Genes and Genomes. Nucleic Acids Res. 2000; 28:27–30. https://doi.org/10.1093/nar/28.1.27 PMID: 10592173

19. Gyorffy B, Lanczky A, Eklund AC, Denkert C, Budczies J, Li Q, et al. An online survival analysis tool to rapidly assess the effect of 22,277 genes on breast cancer prognosis using microarray data of 1809 patients. Breast Cancer Res Treatment. 2010; 123:725–731.

20. Gonzalez Y, Aryal B, Chehab L, Rao VA. Atg7- and Keap1-dependent autophagy protects breast cancer cell lines against mitoquinone induced oxidative stress. Oncotarget. 2013; 5:1526–1537.

21. Go Ma JY, Liu Q, Liu G, Peng S, Wu G. Identification and validation of a robust autophagy-related molecular model for predicting the prognosis of breast cancer patients. Ageing. 2021; 13:13684–16695.

22. Lai J, Chen B, Mok H, Zhang G, Ren C, Liao N. Comprehensive analysis of autophagy -related prognostic genes in breast cancer. J Cell Mol Med. 2020; 24:9145–9153. https://doi.org/10.1111/jcmm.15551 PMID: 32618109

23. Zhou J, Hang D, Jiang Y, Chen J, Han J, Zhou W et al. Evaluation of genetic variants in autophagy pathway genes as prognostic biomarkers for breast cancer. Gene. 2017; 627:549–555. https://doi.org/10.1016/j.gene.2017.06.053 PMID: 28669927

24. Li X, Chen J, Huang H, Liu Z, Wang C, He Y et al. A signature of autophagy -related long non-coding RNA to predict the prognosis of breast cancer. Front Genet. 2021; 12:569318. https://doi.org/10.3389/fgene.2021.569318 PMID: 33796128

25. Jegga AG, Schneider L, Ouyang X, Zhang J. Systems biology of the autophagy-lysosomal pathway. Autophagy. 2011; 7:477–489. https://doi.org/10.4161/auto.7.5.14811 PMID: 21293178

26. Rosenbluth JM, Mays JM, Pino MF, Tang LJ, Pietenpol JA. A gene signature-based approach identifies mTOR as a regulator of p73. Mol Cell Biol. 2008; 28:5951–5964. https://doi.org/10.1128/MCB.00305-08 PMID: 18678646

27. Chen XX, Li ZP, Zhu JH, Xia XT, Zhou H. Systematic analysis of autophagy-related signature uncovers prognostic predictor of acute myeloid leukemia. DNA Cell Biol. 2020; 39:1595–1605. https://doi.org/10.1089/dna.2020.5667 PMID: 32783661
